# China’s three-year health reform program and equity in sanitation improvement: a panel analysis

**DOI:** 10.1186/s12889-015-1364-7

**Published:** 2015-01-31

**Authors:** Xiaolong Li, Yanqing Miao, Wenjing Chen

**Affiliations:** School of Economics and Management, Beijing University of Posts and Telecommunications, Beijing, 100876 China; China National Health Development Research Center, NHFPC, Beijing, 100083 China

**Keywords:** Inequity, Improved sanitation, Three-year health reform program, Central investment, Concentration curve, Concentration index, Absolute concentration index

## Abstract

**Background:**

Accessible improved sanitation is critical to child health, and inequities in improved sanitation can be interpreted as health inequities across socio-economic groups. From 2009 to 2011, the Chinese government invested 4.448 billion yuan for rural sanitation improvement through a 3-year health reform program. This study assesses the inequity of sanitation improvement in rural China from 2003 to 2011 and examines whether the 3-year health reform program promoted equity in sanitation improvement.

**Methods:**

Data from the China Health Statistics Yearbooks of 2004 to 2012 and the National Bureau of Statistics of China were used to create the concentration curve (CC), concentration index (CI), and absolute concentration index (ACI) of improved sanitation. Data of central investment for sanitation improvement in each province of China for 2009, 2010, and 2011 was gained through correspondence and used to create the CC and CI for investment.

**Results:**

Although the CIs of improved sanitation are lower than the CIs of the net income of rural residents, the latter have an obvious downtrend. The CIs of improved sanitation increased from 2003 until 2008 and started to drop in 2009. As a result, by 2011, the CIs of improved sanitation had reached their 2003 levels. The ACI of improved sanitation decreased slightly from 2003 to 2008, but declined sharply from 2009 to 2011. The CIs of central investment for 2009, 2010, and 2011 are negative and the CCs of central investment are above the line of absolute equality, indicating that investments had been concentrated more on poorer provinces and regions.

**Conclusions:**

The equality of rural residents’ net income has been improving each year, whereas equity in sanitation improvement deteriorated from 2003 to 2008. However, equity in sanitation improvement has increased since 2009 due to central investment in sanitation improvement during the 3-year health reform program that benefits low-income areas more. It is clear that the 3-year health reform program played an important role in promoting the level and equity of sanitation improvement.

**Electronic supplementary material:**

The online version of this article (doi:10.1186/s12889-015-1364-7) contains supplementary material, which is available to authorized users.

## Background

Globally, approximately 2.4 million deaths (4.2% of all deaths) [1] could be prevented each year if everyone practised appropriate hygiene and had good, reliable sanitation and drinking water. Most of these deaths were of children in developing countries and primarily resulted from diarrhoea and subsequent malnutrition, as well as from other diseases attributable to malnutrition. Although it is rarely discussed alongside the three major diseases that attract attention from the international public health community, HIV/AIDS, tuberculosis, and malaria, diarrhoea alone kills more children each year than these three combined [2]. The keys to diarrhoea’s control are hygiene, sanitation, and water.

**Figure 1:**
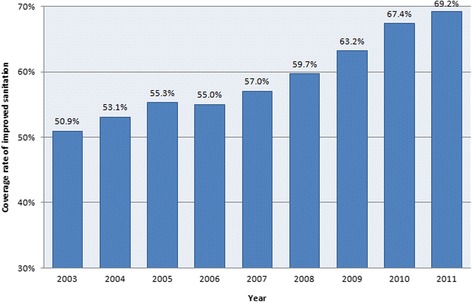
**Coverage rate of improved sanitation in rural China from 2003 to 2011.**

In 2010, Bartram and Caimcross noted that “The household burden weighs most heavily upon the poor, but well-conceived sanitation and water programmes can weaken the link between poverty and disease and so contribute to health equity” [3]. Sanitation improvement in rural China deserves close attention because China is one of the largest developing countries in the world, and it has the largest population in rural areas [4]. The traditional causes of illness, such as infections resulting from poor sanitation and hygiene, are unevenly distributed across China’s diverse geographic landscape as a result of regional differences in economic development, culture and environmental factors [5-7]. The Chinese government has attached special importance to sanitation improvement for more than a decade and has incorporated the improvement of sanitation in rural areas into its national 5-year plan since the 1990s. In 2009, to promote public health service levels and equity, the Chinese government launched a 3-year health reform program. The opinions of the Communist Party of China (CPC) Central Committee and the State Council on widening the medical and health system reform indicated that government input in health services would be gradually increased [8]. The program aimed to alleviate the burden of expensive medical bills on citizens and increase their access to health care services [9]. In addition, the government stated that increasing spending on primary healthcare institutions was one of the five top priorities this plan. According to a 2012 report from the Ministry of Finance, from 2009 to 2011, the government invested approximately 1409.9 billion yuan (approximately US$ 206 billion) in health care, and 44% of these funds were allocated for primary healthcare institutions [10]. In these 3 years, the Chinese government invested a total of 4.448 billion yuan for rural sanitation improvement. The main focus of the sanitation improvement program is helping rural residents build and use an improved sanitation system that requires secure access to a hygienic latrine, as well as treatment and safe disposal of sewage or wastewater. Large inputs brought high returns, and by the end of 2011, China had built 180,185,000 improved sanitation facilities in rural areas, of which 10,289,000 were completed in 2011 [11]. In addition, the coverage rate of improved sanitation in rural China reached 69.2% in 2011 [12]. Figure 1 shows that the domestic coverage rate of improved sanitation in rural areas from 2003 to 2011 increased year by year.

In terms of the disparity in sanitation coverage rates among different regions, by the end of 2011, the highest was 98% in Shanghai (rural per capita net income of 16,053.8 yuan in 2011) and the lowest was 40.9% in Guizhou Province (rural per capita net income of 4,145.4 yuan in 2011) [13]. These data show that the sanitation coverage rate varies greatly among different regions due to substantial gaps in economic development levels, efficiency of government execution, and other factors. However, research on the equity of sanitation improvement in rural China is sparse, and the existing research does not include discussion of the distribution of central investment in sanitation improvement during the 3-year health reform program from 2009 to 2011.

Among the different measures used for assessing inequity of water and sanitation improvement, the most common is the concentration index (CI) [14-17]. The relative CI does not take into account the level of a certain variable within a population, only how much it varies. Some researchers use the absolute concentration index (ACI) to measure public health condition and its equity simultaneously [18] because both public health condition and its equity are vital in public health policy. However, only a few studies focusing on ACIs are available [19-21]. To date, no researchers have used ACI to study sanitation improvement and related problems.

Therefore, we used the provincial panel data to assess inequity in sanitation improvement with an approach of calculating the CI and ACI of improved sanitation and examine whether the 3-year health reform program promoted equity in sanitation improvement. A number of studies have revealed that income and government intervention are major determinants of sanitation improvement [22-24], so we also calculated the CI of rural residents’ net incomes as a reference and studied the distribution of central investment in 3-year health reform program from 2009 to 2011.

## Methods

### Data resources

The gross number of households and households with improved sanitation in rural China are from the China Health Statistics Yearbooks of 2004, 2005, 2006, 2007, 2008, 2009, 2010, 2011, and 2012. Peking Union Medical College Press publishes the China Health Statistics Yearbook annually, and the yearbook reflects China’s annual health career development situation and resident health status information. The yearbook is a collection of national health development in 31 provinces, autonomous regions and municipalities directly under the Central Government and includes statistics for the current level of residents’ health. Every yearbook incorporates contents for the end of the previous year. For example, the *China Health Statistics Yearbook 2012* incorporates contents for the end of 2011. The yearbook is based on information from multiple sources, and the data for sanitation is from the annual health statistics report.

Rural residents’ net incomes from 2003 to 2011 are from the China Statistical Yearbook. The amount of central investment for sanitation improvement in each province in 2009, 2010, and 2011 was obtained through correspondence from the Patriotic Public Health Campaign Committee of each province, which is in charge of specific implementation of the national health program, including sanitation improvement. Table 1 shows the results of this correspondence. The data in this study covered all provinces in China except Tibet.

**Table 1:** **Central investment for sanitation improvement in each province in 2009, 2010, and 2011 (in millions of yuan)**

**Province**	**2009**	**2010**	**2011**
Gansu	55.16	68	55
Guizhou	66	25	25
Qinghai	26.48	15	15
Yunnan	63.4	64	95
Shaanxi	80.055	76.5	110
Xinjiang	47.2	48	95
Guangxi	18.4	110.8	75
Ningxia	26.48	8	25
Shanxi	63.92	40	25
Sichuan	87.28	56	100
Chongqing	75.44	80	115
Anhui	70	64	80
Hainan	33.2	20	15
Henan	40	40	60
Hunan	14	138.5	100
Neimenggu	61.84	48	55
Hubei	100	97.2	75
Jiangxi	69	64	100
Hebei	67.86	52	40
Heilongjiang	60.1	40	60
Jilin	61	56	35
Liaoning	8.75	44.52	33
Shandong	20	20	20
Fujian	27.27	48	9
Guangdong	25.91	45	42.76
Jiangsu	75	75	75
Tianjin	12.12	0	12.12
Zhejiang	28.096	27	30
Beijing	12.12	21	20
Shanghai	0	0	0

### Concentration Curve (CC)

The construction approach to the CC is similar to the approach for building a Lorenz curve. The horizontal axis is the cumulative proportion of the population and is ranked by socioeconomic status, beginning with the least advantaged. The vertical axis is the cumulative distribution of a parameter such as improved sanitation or central investment [25-28]. Compared with the curve of absolute equality, the CC will deviate from the right angle bisector if the distribution of the indicator is uneven among populations of different socioeconomic status: the more uneven the indicator, the more severe the deviation. The socioeconomic status of the population upon which the indicator is concentrated can also be interpreted from the shape of the curve.

The CC is not always a convex curve that monotonically increases because the cumulative parameter of vertical axis may be sorted differently from the parameter of the horizontal axis.

### Concentration Index (CI)

The CI is defined as twice the area between the CC and the line of equality. Thus, when CC coincides with the line of equality, the CI equals 0. However, when the CC is above the equality line, the CI takes a negative value, and when the CC is below the equality line, the CI takes a positive value. The CI is bound between −1 and +1 [29].

In our study, the CI summarizes the CC and quantifies the degree of economic-related inequality in improved sanitation. Broadly speaking, the CI shows the relationship between the situation of sanitation improvement and economic status; it indicates the direction of the relationship and its magnitude echoes both the strength of the relationship and the degree of variability in the distribution of improved sanitation.

Suppose we have K provinces, and they are sorted by income from low to high. There are *n*_1_, *n*_2_, *n*_3_ … *n*_*K* − 1_, *n*_*K*_ households in these provinces respectively and *σ*_1_, *σ*_2_, *σ*_3_ … *σ*_*K* − 1_, *σ*_*K*_ coverage rate of improved sanitation. Then, the total households are n = n_1_ + n_2_ + n_3_ + … + n_K ‐ 1_ + n_K_, with the weight corresponding to *ith* province: ${\mathrm{w}}_{\mathrm{i}}=\frac{{\mathrm{n}}_{\mathrm{i}}}{\mathrm{n}}\operatorname{}$ .

We used integration to measure twice the area between the CC and the line of equality. The *CI* in our model is:

1 $$ \mathrm{C}\mathrm{I}=\frac{2}{\upmu}{\displaystyle {\sum}_{\mathrm{i}=1}^{\mathrm{K}}{\mathrm{y}}_{\mathrm{i}}{\mathrm{R}}_{\mathrm{i}}\hbox{-} }1 $$

Where μ is the coverage rate of improved sanitation of China and can be computed as:

2 $$ \upmu ={\displaystyle {\sum}_1^{\mathrm{n}}{\mathrm{w}}_{\mathrm{i}}{\upsigma}_{\mathrm{i}}} $$

*R*_i_ is the relative rank of the *ith* province, defined as:

3 $$ {\mathrm{R}}_i=\frac{1}{2}\cdotp \frac{{\displaystyle {\varSigma}_{j=1}^{i-1}\;{n}_j+{\varSigma}_{\mathrm{j}=1}^i{n}_j}}{n} $$

and indicating the cumulative proportion of the population up to the midpoint of each group interval [26].

*y*_*i*_ is the proportion of improved sanitation in *ith* province to the total households and can also be treated as the synthesis score, which can be computed as:

4 $$ {\mathrm{y}}_{\mathrm{i}}={\mathrm{w}}_{\mathrm{i}}{\upsigma}_{\mathrm{i}} $$

To calculate integration, we must first calculate the differential. Then, the area between the absolute equality line and the *CC* line can be divided into K small areas, and each area can be treated as a trapezoid (approximately). R_i_ is the half of the sum of *ith* trapezoid’s upper base and lower base.

### Absolute Concentration Index (ACI)

The ACI of health is obtained by multiplying the health CI with the mean of health variable [27], while the ACI of improved sanitation is defined as follows:

5 $$ ACI=C\cdot \left(1-\mu \right) $$

where *C* is the improved sanitation CI and μ is the coverage rate of improved sanitation. The value of ACI decreases when imparity in the improved sanitation coverage rate for all the provinces decreases or the improved sanitation coverage rates for each province increases at the same time. Therefore, the ACI of improved sanitation evaluates equality and progress in sanitation improvement simultaneously.

## Results

### CCs of central investment, improved sanitation, and net income of rural residents

**Figure 2:**
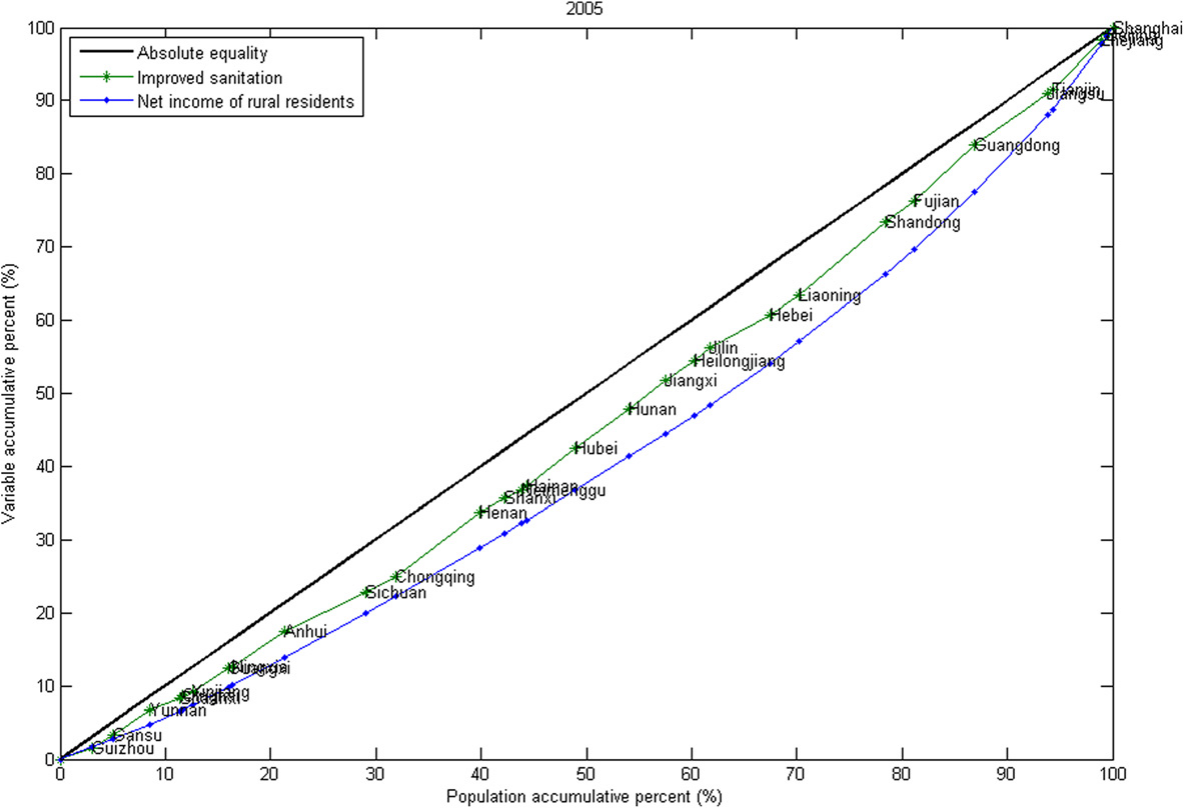
**Concentration curves for 2005.**

**Figure 3:**
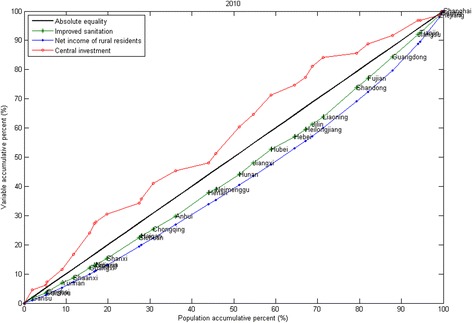
**Concentration curves for 2010.**

Figure 2 shows the CCs for 2005. The CCs for the other years from 2003 to 2008 are all similar to the CCs for 2005 and are included in the Additional file 1. Figure 3 shows the CCs for 2010. The CCs for 2009 and 2011 are similar to the CCs for 2010 and are included in the Additional file 1. For every year, the CC of improved sanitation and the CC of net income of rural residents both lie below the line of absolute equity, but the CC of improved sanitation lies between the CC of net income of rural residents and the line of absolute equality. Based on this information, it can be interpreted that from 2003 to 2011, income and improved sanitation were more concentrated in regions with good economic statuses. However, the inequality in improved sanitation was less than the inequality in income for the years 2003 to 2011. From 2009 to 2011, during the 3-year health reform program, the CCs of central investment are all above the line of absolute equality. This shows that the distribution of central investment benefited the disadvantaged groups more than groups that were not disadvantaged.

### CIs of central investment, improved sanitation, and net income of rural residents and ACI of improved sanitation

**Figure 4:**
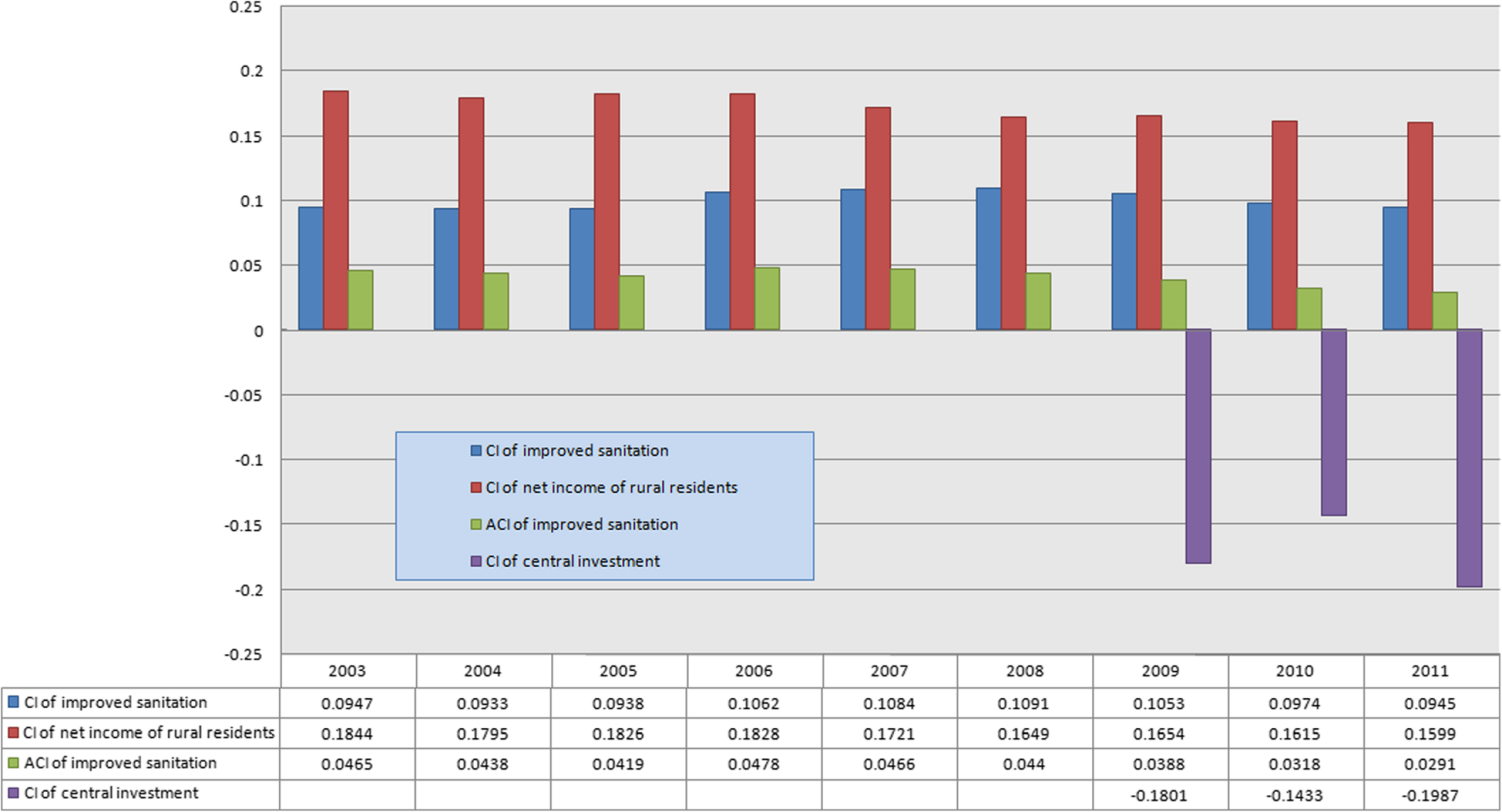
**CIs and ACI from 2003 to 2011.**

Figure 4 presents the three CIs and the ACI. From 2003 to 2011, there is an obvious downtrend of the CI for net income of rural residents. The CI declines from 0.1844 in 2003 to 0.1599 in 2011, indicating that the inequality of rural residents’ income decreased year by year. In the meantime, although it was lower than the CI for rural residents’ net income, the CI for improved sanitation had been increasing from 2003 to 2008. However, it started to drop in 2009, and by 2011, the CI for improved sanitation had reached the same level as the CI for 2003.

The CIs of central investment for 2009, 2010, and 2011 are negative, indicating that investments had been more concentrated on poorer provinces and regions. The ACI of improved sanitation had a slight drop from 2003 to 2008, but declined sharply from 2009 to 2011. This means that the improved sanitation coverage rates for each province are increasing simultaneously, and thus the imparities among the provinces are narrowing.

### Change in improved sanitation coverage in east, west, and central China

**Figure 5:**
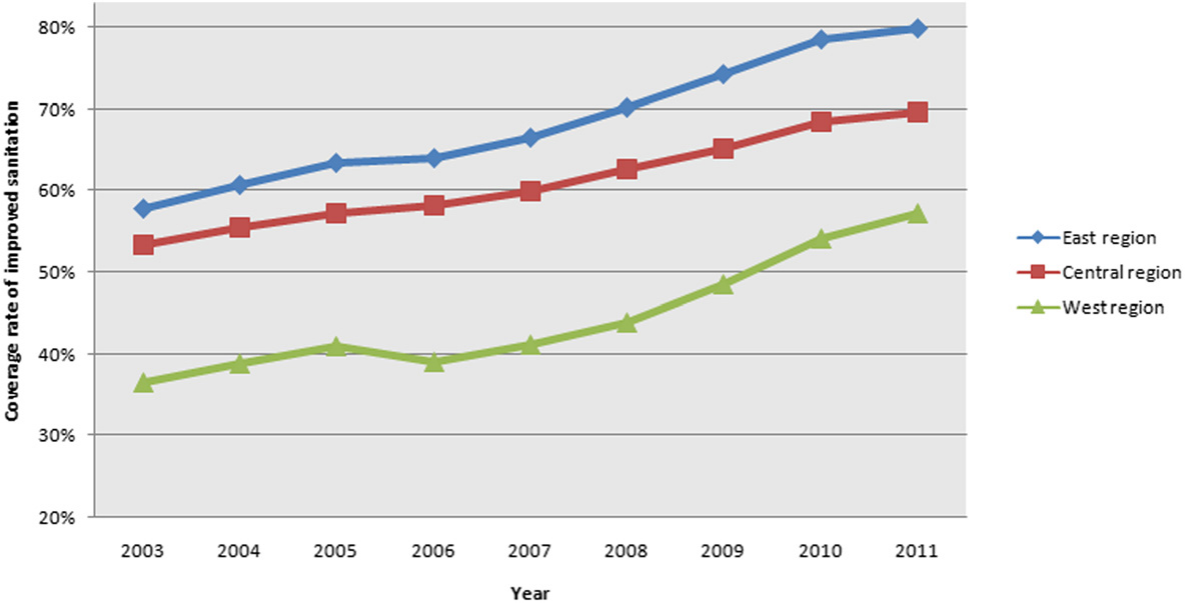
**Coverage of improved sanitation in different regions of China.**

Figure 5 indicates that improved sanitation coverage rates of east, west, and central China are increasing each year. However, from 2003 to 2008, there is a marked difference in improved sanitation coverage rate between west China and central China, as well as between west China and east China. In 2009, the difference began to narrow, and this is one main reason that the CI and ACI of improved sanitation had been decreasing since 2009.

## Discussion

Due to limited resources, determining how to make a fairer allocation of public resources often creates a dilemma for policymakers [30]. One of the reasons for this dilemma is the complexity involved in defining “equity” [31]. One element of defining equity is making a “value judgment” [32]. In recent years, many public service scholars have stated that the core ideology of equity or fairness indicates that input should vary as the needs of the population vary. In that case, the goal of equity is defined as equally attainable, which means public resources should be of the same attainability to people with the same needs for public services. When trying to achieve equity in public services or resource distribution, most industrialized countries would refer to the concept of “equally attainable”. We therefore evaluated the equity in sanitation improvement of China by calculating the CI of improved sanitation, CI of central investment, CI of rural residents’ net income, and ACI of improved sanitation.

In terms of equity, judging from the CIs of improved sanitation, central investment, and rural residents’ net income, the equality of rural residents’ net income has been improving year by year. Meanwhile, equity in sanitation improvement deteriorated from 2003 to 2008. We believe the main cause of this deterioration is that the willingness of rural residents in east China to pay for improved sanitation is higher than that of rural residents central and west China [33]. Therefore, the disparity of the ratio of improved sanitation between the provinces in east China and the provinces in central and west China would increase without intervention. However, equity had become better starting in 2009, likely due to the central investment during the 3-year health reform program in sanitation improvement that benefits low-income populations more. The CIs of central investment from 2009 to 2011 are all negative, and the CCs are all above the line of absolute equality. On the other hand, west China, whose economy lags behind the economies of east and central China, had remarkably improved sanitation coverage as well. Some scholars indicated that the contributions of the main determinants of sanitation improvement (including economic status, minority status, education, and temperature) to the inequality of sanitation improvement in China from 2003 to 2011 did not change substantially [34]. The 3-year health reform program therefore clearly played an important role in promoting equity in sanitation improvement. However, although the equity of sanitation improvement increased year by year as a result of the 3-year health reform program, the situation may not be quite so optimistic from an overall perspective because equity in sanitation improvement in 2011 was the same as in 2003, although the equity of net income of rural residents had been increased.

Calculating ACIs, and not only relative CIs (as has been done in most previous studies), provides information on both the level and distribution of improved sanitation [18]. This is important because the aim of sanitation improvement policy, and all public health policy, is twofold: to improve the level and attain equity. The ACI thus provides a performance measure for the overarching aim of sanitation improvement policy.

Equity in sanitation improvement is difficult to measure, so we put forward a method to calculate the CI of improved sanitation that combines net income of rural residents with central investment in sanitation improvement to evaluate equity. It is also the first time a study has systematically evaluated sanitation improvement during these years in China. However, sanitation improvement is in fact influenced by many other factors, including climate and nationality [23,34,35]. How these factors influence the equity of sanitation improvement and any bottlenecks in further equity improvement must be included in further study. In addition, in this study, we only considered the variables that are easy to quantify, such as the ratio of improved sanitation and central investment. However, it is also important to consider variables such as knowledge, attitude and practice (KAP) which cannot be arrived at directly. We believe there is a marked effect of disparity in KAP on sanitation improvement in China and can strongly influence the ratio of improved sanitation. In addition, our study is based on provincial panel data for China, which means that the imparities we discussed are among provinces, not within each province. To determine provincial imparities, more detailed data are needed, such as data at the municipal level or county level.

The ultimate goal of evaluating equity in sanitation improvement is to eliminate inequity, which requires determining what caused the inequity so the bottleneck and any constraints on sanitation improvement can be removed individually. This is the common perspective of many researchers and related departments and an important research goal.

## Conclusion

Using the approach of CI and ACI, we found that the equality of rural residents’ net income has been improving each year, whereas equity in sanitation improvement deteriorated from 2003 to 2008. However, equity in sanitation improvement has increased since 2009 due to central investment in sanitation improvement during the 3-year health reform program, which benefits low-income areas more than higher-income areas. Based on this study, it is clear that the 3-year health reform program played an important role in promoting the level and equity of sanitation improvement in China.

## Additional file

Additional file 1:
**Concentration curves for 2003 to 2012.**
