# Supplementary figures and images for: China’s three-year health reform program and equity in sanitation improvement: a panel analysis
Source: BMC Public Health. 2015 Jan 31;15:38. doi: 10.1186/s12889-015-1364-7 (PMC4323256; doi:10.1186/s12889-015-1364-7)

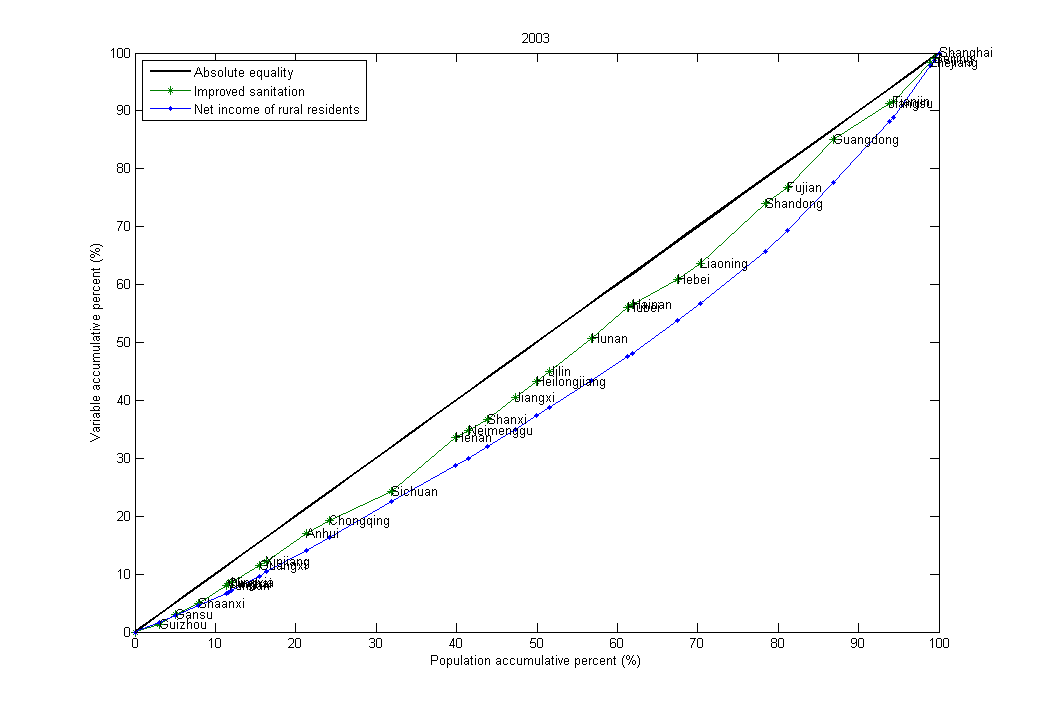

Supplement: Additional file 1: — Concentration curves for 2003 to 2012. [file 12889_2015_1364_MOESM1_ESM.zip › supplementary material/Concentration curves for 2003.png]

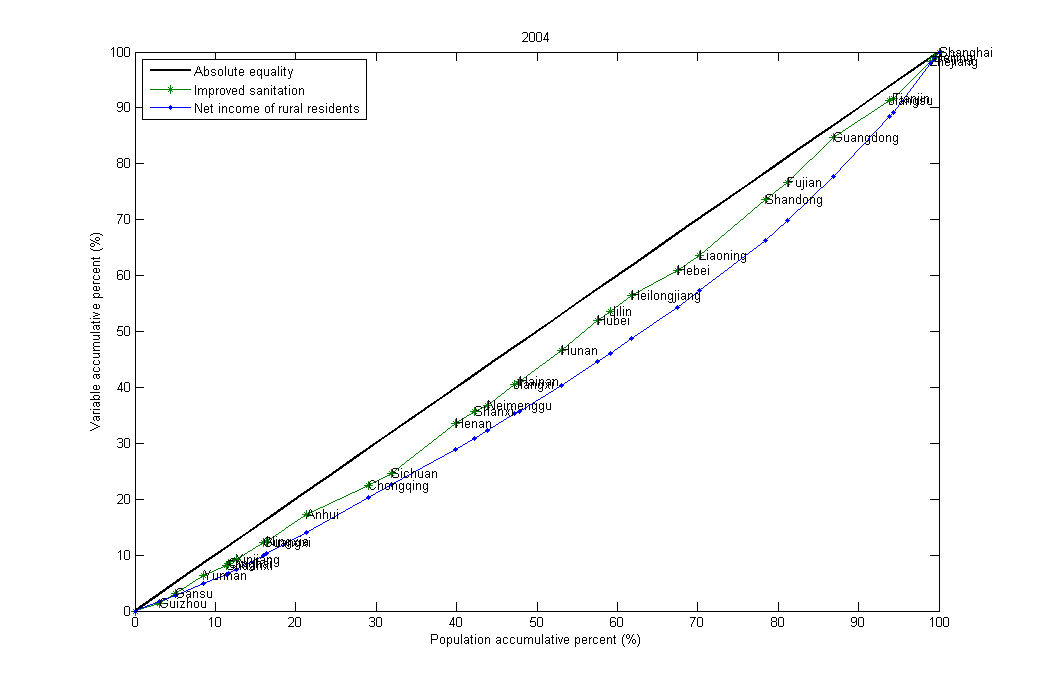

Supplement: Additional file 1: — Concentration curves for 2003 to 2012. [file 12889_2015_1364_MOESM1_ESM.zip › supplementary material/Concentration curves for 2004.png]

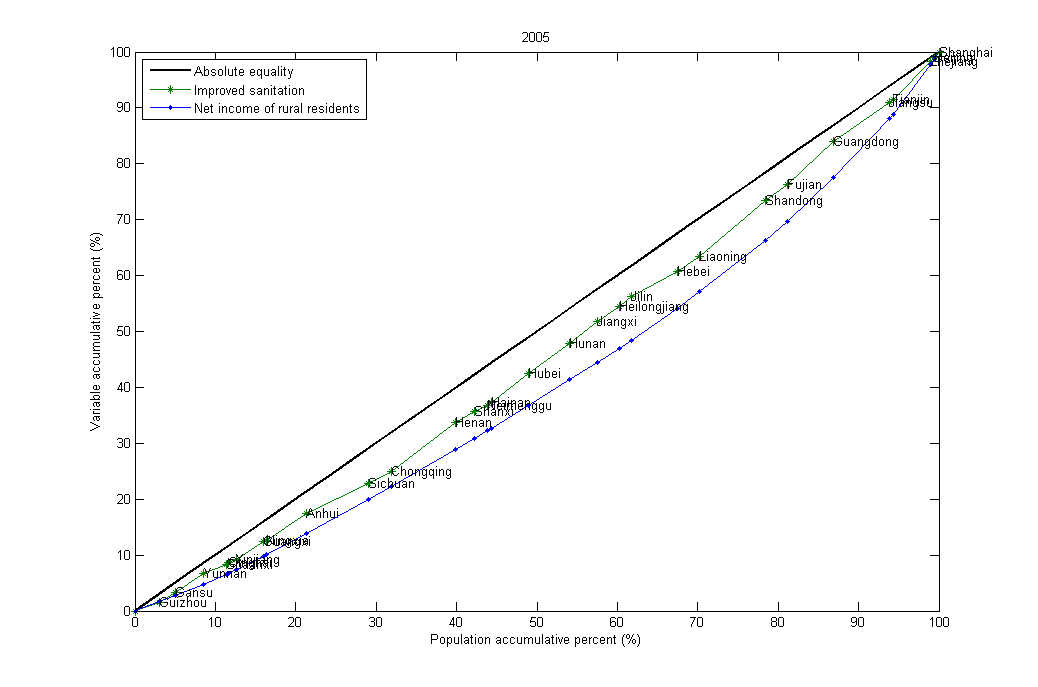

Supplement: Additional file 1: — Concentration curves for 2003 to 2012. [file 12889_2015_1364_MOESM1_ESM.zip › supplementary material/Concentration curves for 2005.png]

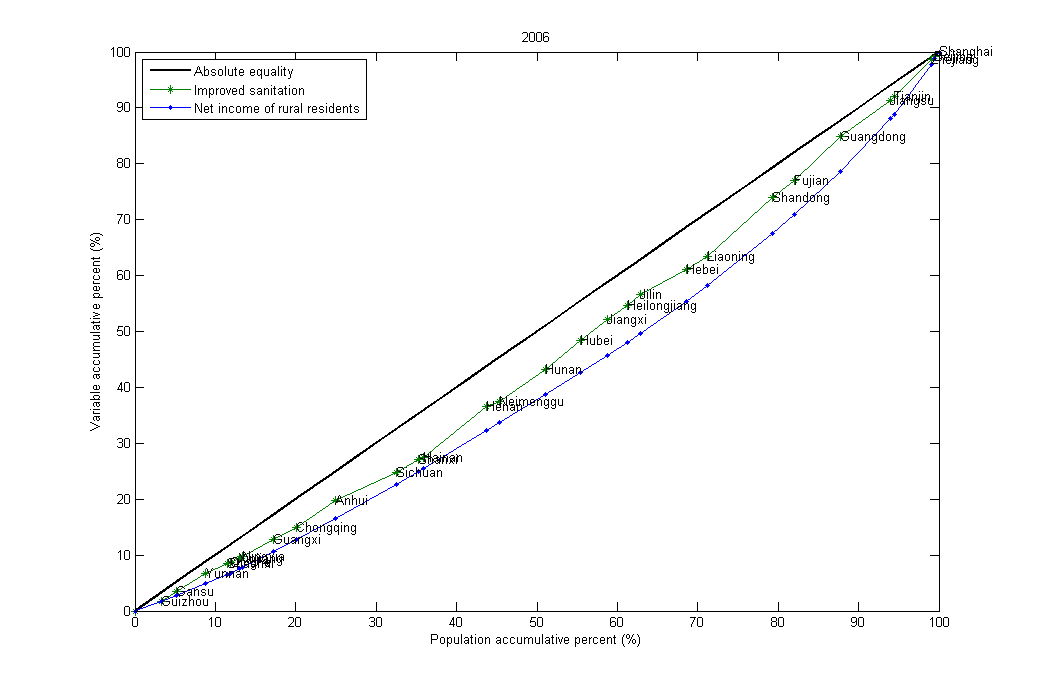

Supplement: Additional file 1: — Concentration curves for 2003 to 2012. [file 12889_2015_1364_MOESM1_ESM.zip › supplementary material/Concentration curves for 2006.png]

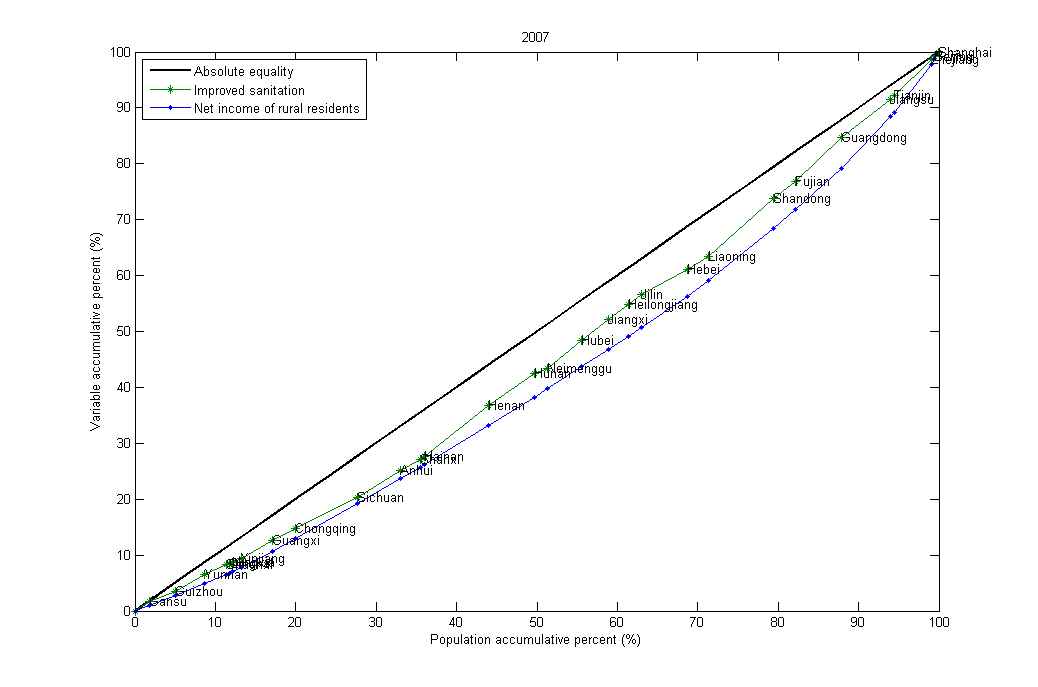

Supplement: Additional file 1: — Concentration curves for 2003 to 2012. [file 12889_2015_1364_MOESM1_ESM.zip › supplementary material/Concentration curves for 2007.png]

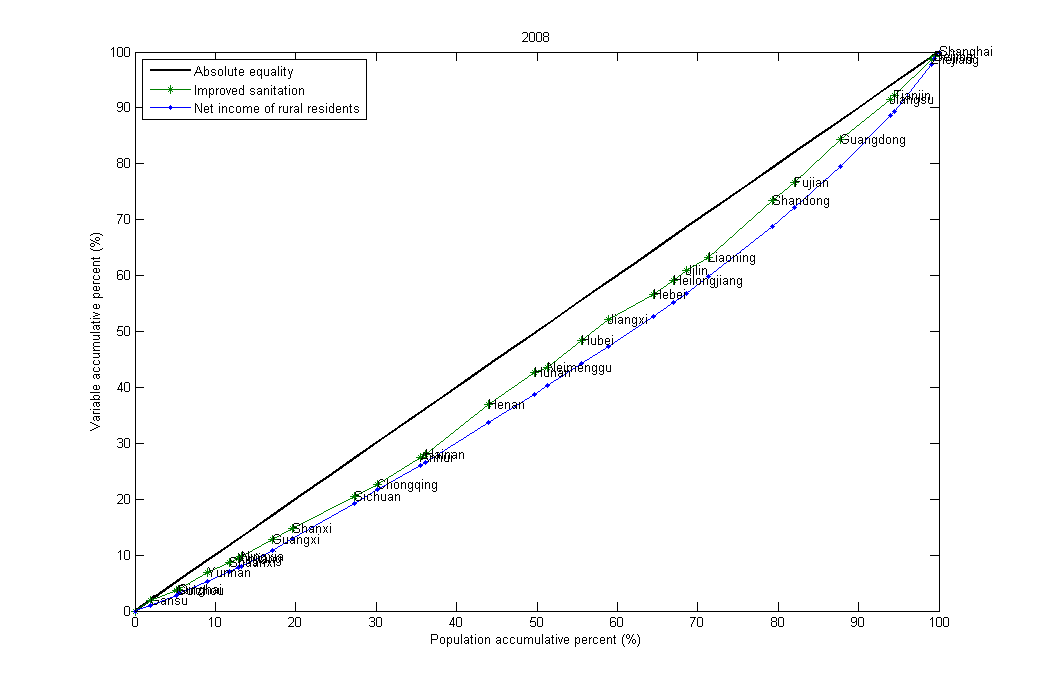

Supplement: Additional file 1: — Concentration curves for 2003 to 2012. [file 12889_2015_1364_MOESM1_ESM.zip › supplementary material/Concentration curves for 2008.png]

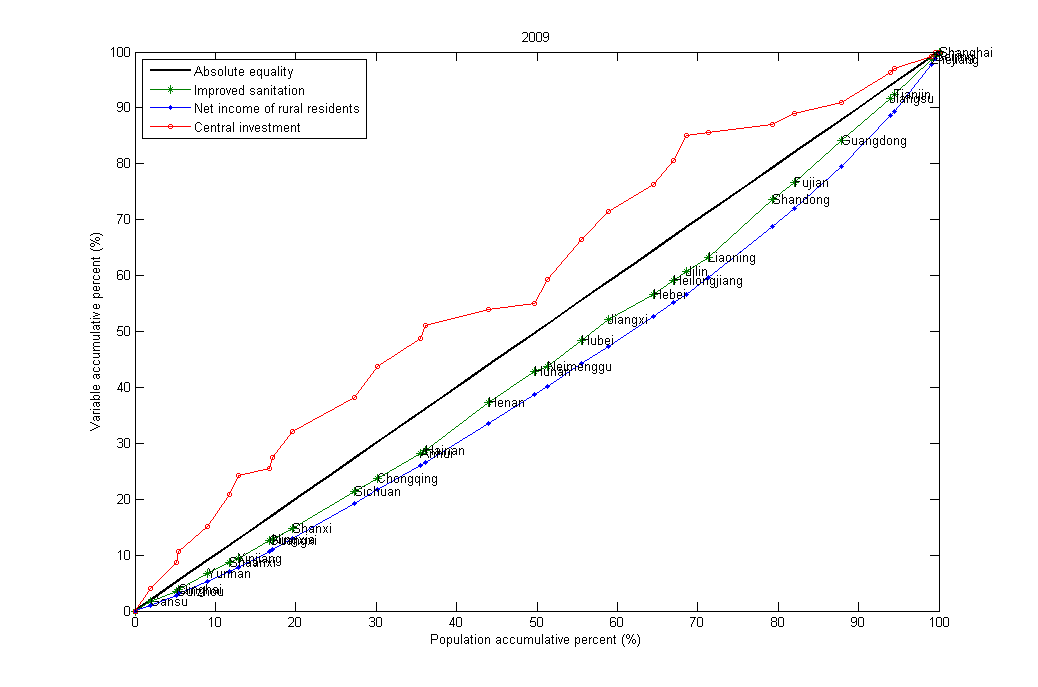

Supplement: Additional file 1: — Concentration curves for 2003 to 2012. [file 12889_2015_1364_MOESM1_ESM.zip › supplementary material/Concentration curves for 2009.png]

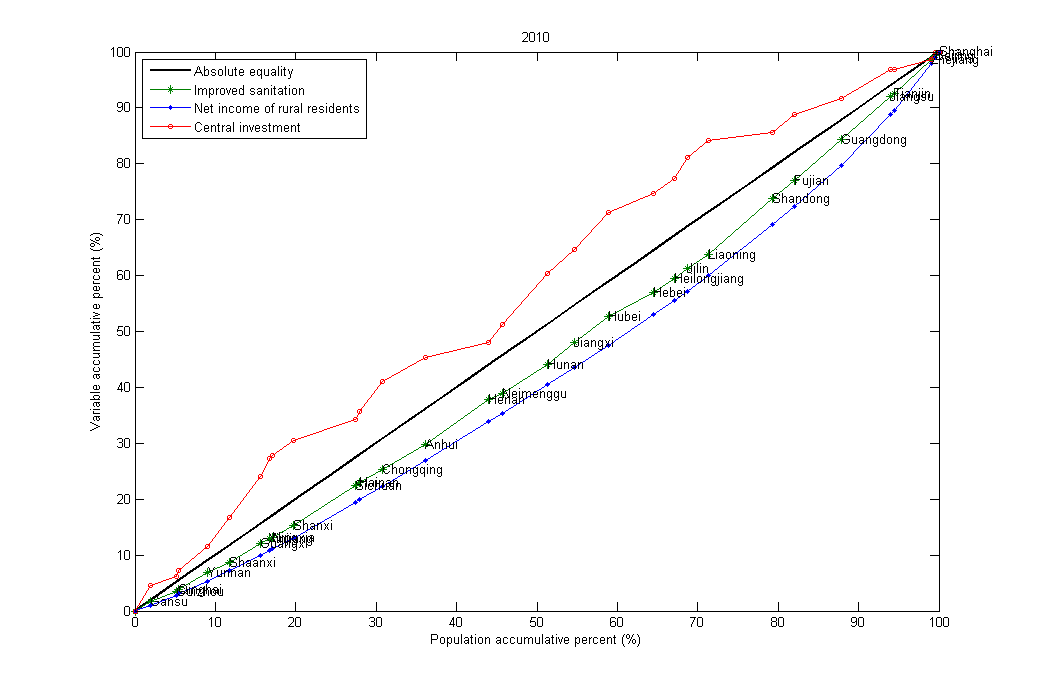

Supplement: Additional file 1: — Concentration curves for 2003 to 2012. [file 12889_2015_1364_MOESM1_ESM.zip › supplementary material/Concentration curves for 2010.png]

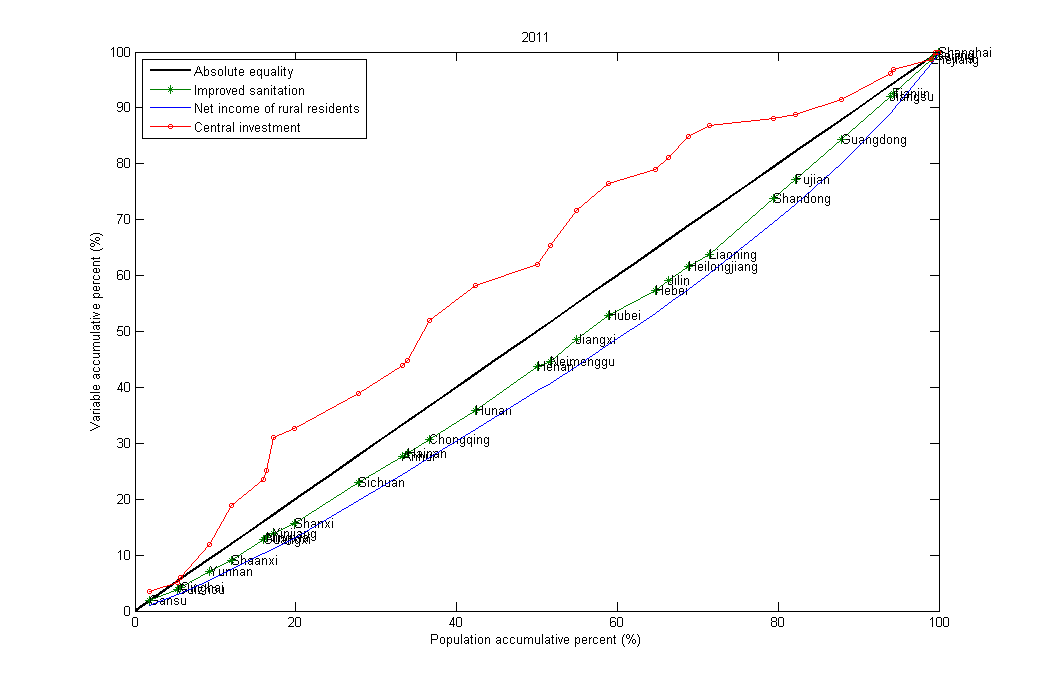

Supplement: Additional file 1: — Concentration curves for 2003 to 2012. [file 12889_2015_1364_MOESM1_ESM.zip › supplementary material/Concentration curves for 2011.png]
